# Association Between Influenza Vaccine and Immune Thrombocytopenia: A Systematic Review and Meta-Analysis

**DOI:** 10.3390/vaccines12111298

**Published:** 2024-11-20

**Authors:** Zhicai Liu, Jing Wang, Zhaojun Lu, Yuyang Xu, Jian Du, Jiayin Han, Xuechao Zhang, Yan Liu

**Affiliations:** 1 Department of Public Health, Medical School of Ningbo University, Ningbo 315211, China; 2311140299@nbu.edu.cn; 2 Department of Infectious Disease Control and Prevention, Hangzhou Center for Diseases Control and Prevention (Hangzhou Health Supervision Institution), Hangzhou 310021, China; wangjing@hzcdc.com.cn; 3 Department of Expanded Program on Immunization, Hangzhou Center for Diseases Control and Prevention (Hangzhou Health Supervision Institution) Hangzhou 310021, China; luzj@hzcdc.com.cn (Z.L.); xuyuyang@hzcdc.com.cn (Y.X.); dujian@hzcdc.com.cn (J.D.); hanjiayin@hzcdc.com.cn (J.H.); xchzhang@hzcdc.com.cn (X.Z.)

**Keywords:** Immune thrombocytopenia, purpura, influenza, vaccination, meta-analysis

## Abstract

Background: Immune thrombocytopenia (ITP) is an uncommon but serious adverse reaction after vaccination. However, its association with vaccines other than the measles–mumps–rubella vaccine remains debatable. This study aimed to analyze ITP cases following influenza vaccination and assess any potential association. Methods: We performed a systematic search of the Web of Science, Embase, and PubMed databases from their inception to 15 April 2024. Cases were characterized qualitatively, and relative risk was assessed using either fixed or random models. Results: A total of 24 studies were analyzed, including 16 patients from 14 case reports. Patients averaged 56.7 years old, half were female, and ten patients had a history of prior illness. The mean time between vaccination and diagnosis was 13.3 days. Treatment primarily involved corticosteroids or intravenous immunoglobulin, with most recovering within a month. The pooled odds ratio for ITP post-influenza vaccination was 0.94 (95%CI: 0.85–1.03). Subgroup analyses conducted according to the study design and vaccine type did not reveal any significant results. Conclusion: No evidence of an association between influenza vaccination and ITP was found. Further observational studies are required to verify this relationship.

## 1. Introduction

Seasonal influenza, typically referred to as the flu, is a highly contagious viral infection that primarily targets the respiratory system. It can result in various mild-to-severe symptoms, particularly in vulnerable populations such as older adults, young children, pregnant individuals, and those with weakened immune systems [1]. Influenza causes approximately 1 billion infections yearly, of which 3–5 million are serious illnesses [2]. Vaccination is crucial for disease prevention and is one of the most cost-effective public health measures available. Annual influenza vaccination significantly reduces influenza-related morbidity and mortality. Influenza vaccines have beneficial effects on patients with chronic diseases, cardiovascular diseases, and immunocompromised patients, such as reducing the risk of acute respiratory infections in patients with chronic obstructive pulmonary disease and hospitalization in patients with heart failure [3,4]. There are three types of influenza vaccines: inactivated, recombinant, and live attenuated. The exact type of vaccination is determined based on the patient’s age and physical condition [5].

Vaccination can occasionally lead to adverse reactions, such as herpes zoster and meningitis from the varicella vaccine and allergic reactions from DTaP or Tdap [6]. These reactions are less frequent and severe than those caused by natural infections; however, they are still of concern. Immunization-related autoimmune diseases are among the most worrisome adverse reactions [7]. Aside from the rare associations of the measles–mumps–rubella (MMR) vaccine with immune thrombocytopenia and that of the influenza vaccine with Guillain–Barre syndrome (GBS), a definitive relationship between vaccines and the development of autoimmune diseases remains unproven [8]. Similar to other vaccines, the influenza vaccine can cause local or systemic adverse effects, commonly manifesting as fever, injection site pain, erythema, swelling, and induration. The attenuated influenza vaccine may also lead to headache, nasal congestion, and sore throat [9]. In specific seasons, the influenza vaccine has been associated with an increased risk of GBS, a rare neurological disorder [10]. Additionally, serious adverse effects such as stroke, encephalitis, peripheral neuropathy, and immune thrombocytopenia have been reported following influenza vaccination [11].

Immune thrombocytopenia (ITP), formerly known as idiopathic thrombocytopenic purpura, is an autoimmune disorder that is primarily defined by the exclusion of other causes of low platelet counts (<100 × 10^9^/L). This condition arises from the immune system mistakenly targeting and destroying platelets, leading to an increased risk of bleeding and related complications. Clinical signs include skin hemorrhages, such as purpura, rash, and bruises, and mucosal hemorrhage, such as epistaxis and gingival bleeding. However, in severe cases, bleeding can extend to the subarachnoid or intracerebral regions, the lower gastrointestinal tract, or other internal areas [12]. The prevalence of ITP is estimated to range from approximately 1.6–3.9 cases per 100,000 person-years. Additionally, epidemiological studies have shown that the overall average incidence of this disorder tends to increase with age and is more prevalent among women [13,14].

ITP is typically idiopathic; however, it can also be secondary to autoimmune diseases, infections, cancers, and drugs [15]. Regarding drug-related ITP, approximately half of the cases can be attributed to vaccines, and the mechanism for the increased risk of ITP after vaccination appears to be the same as that for the induction of antiplatelet autoantibodies by microbial infections [16]. The MMR vaccine is currently the only vaccine proven to be associated with thrombocytopenia, with an estimated 1 case per 20,000–40,000 doses [17]. Numerous surveillance data and cases have also reported the occurrence of ITP following other vaccinations, including influenza, Bacillus Calmette–Guerin, COVID-19, encephalitis B, hepatitis B virus, polio, diphtheria–tetanus–acellular pertussis, varicella, and enterovirus [18,19,20,21]. Therefore, ITP is receiving more and more attention. However, as the most used vaccine globally, no conclusive causal link exists between the influenza vaccine and ITP. Grimaldi-Bensouda et al. and Garbe et al. conducted separate case–control studies to evaluate the potential association between the influenza vaccine and ITP; however, they had contradictory results [22,23]. Therefore, whether influenza vaccination truly increases the risk of ITP or whether the reported cases are coincidental remains unclear. In addition, studies have reported vaccine hesitancy in the general population towards influenza vaccination due to concerns and misconceptions about the risk of adverse reactions [24]. Collecting, analyzing, and evaluating data on suspected abnormal vaccination reactions after influenza vaccination can effectively improve public confidence in the safety of influenza vaccines and provide a reference basis for developing and implementing immunization strategies.

Thus, to clarify the relationship between ITP and influenza vaccination, a systematic review and meta-analysis was performed after summarizing relevant epidemiological studies.

## 2. Materials and Methods

### 2.1. Data Sources

This study was conducted in strict accordance with the Preferred Reporting Items for Systematic Reviews and Meta-Analyses guidelines to ensure transparency and methodological rigor in the research process. Our study was registered in INPLASY (ID: INPLASY2024100107).

We performed a comprehensive search of all relevant articles on the connection between ITP and influenza vaccination, published up to 15 April 2024, in the Embase, Web of Science, and PubMed databases. The main search terms were “Purpura, Thrombocytopenic, Idiopathic”, “Immune Thrombocytopenia”, “Immune Thrombocytopenic Purpura”, “Thrombocytopenic Purpura”, “Idiopathic Thrombocytopenic Purpura”, “influenza”, “flu”, “vaccin*”, and immuni*”. Furthermore, references to the retrieved articles were checked for additional relevant articles.

### 2.2. Eligibility Criteria

The inclusion criteria for the studies were defined as follows: (1) case reports of ITP following influenza vaccination and (2) observational studies that reported an odds ratio (OR), incidence rate ratio (IRR), or risk ratio (RR) with a 95% confidence interval (CI) of ITP after influenza vaccination. We excluded (1) duplicate articles; (2) reviews, conference abstracts, or comments; and (3) papers without data or full text.

### 2.3. Study Selection

All search results were imported into the EndNote X9 software for deduplication and reference management. The remaining articles underwent an initial screening based on their titles and abstracts, and full-text searches were performed on those deemed eligible. Studies were ultimately included after screening by two independent researchers who strictly followed the predefined inclusion and exclusion criteria. Inconsistent results were addressed by mutual discussion or consultation with a third researcher.

### 2.4. Data Extraction and Quality Assessment

Two researchers independently completed data collection using a standardized data extraction form. The following critical information was extracted from the case reports: first author, year of publication, patient age and sex, medical history, number of doses, platelet count before and after vaccination, time from vaccination to diagnosis, time to recovery from ITP, presentation, treatment strategy, and outcomes. For observational studies, we extracted the first author, study design, study area, age, vaccine type, and OR, RR, or IRR with 95% CI. The quality assessment of the observational studies included in this analysis was conducted using the Newcastle–Ottawa Scale (NOS). Scores on the NOS range from 0 to 9, with ≥7 being considered high quality and included in this study. Inconsistent results were addressed by mutual discussion or consultation with a third researcher.

### 2.5. Data Synthesis

We qualitatively analyzed the case reports. A meta-analysis was performed using Review Manager 5.3 and Stata 15.0 (Stata Corp, College Station, TX, USA). The pooled effect size is expressed as the OR of ITP with influenza vaccination compared with no vaccination, and the corresponding forest plot was obtained. As this outcome is rare in the population, ORs can be considered approximations of RRs or IRRs. Cochran’s Q and I^2^ statistics were employed to assess the heterogeneity among the included studies, and statistical heterogeneity was defined as I^2^ > 50%. When I^2^ was > 50%, we used a random-effects model to amalgamate the effect sizes; conversely, a fixed-effects model was employed. Subgroup analyses were performed according to study design and vaccine type. Sensitivity analyses were performed to test the robustness of the findings using a study-by-study approach to exclude individual studies. Funnel plots, in conjunction with Begg’s test and Egger’s test, were implemented to assess potential publication bias.

## 3. Results

### 3.1. Search Results

**Figure 1:**
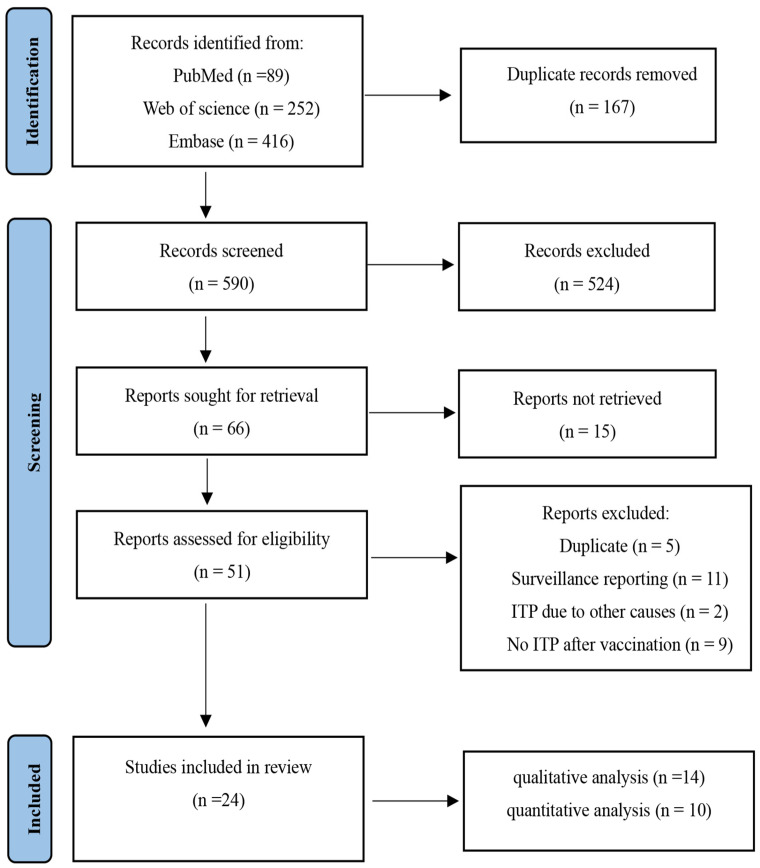
Flow chart of the included studies.

The literature search and selection process is illustrated in Figure 1. Overall, 757 studies were retrieved from three databases, 167 of which were identified as duplicates. Following the screening of the titles and abstracts, 524 articles were disqualified. Finally, 24 studies, all in English, were deemed eligible for inclusion through a full-text screening.

### 3.2. Baseline Characteristics of the Included Studies

Among the included studies, fourteen, three, four, and three were case reports, case–control studies, cohort studies, and self-controlled case series, respectively. The 14 case reports collectively involved 16 patients. The mean age at initial presentation was 56.7 (a range of 3–88) years, with an equal sex distribution among the patients. Ten patients had a history of prior illness. Three cases occurred after the first and third doses of the influenza vaccination, respectively, and one occurred after the second dose. Platelet counts were markedly reduced after influenza vaccination in all patients. The mean interval from the vaccination to ITP onset was 13.3 (a range of 2–35) days. Most patients recovered within a month of diagnosis, with two taking approximately 5 months and a year to recover, respectively. Fifteen patients presented with cutaneous mucosal bleeding, including gingival bleeding, purpura, petechiae, rash, epistaxis, hemoptysis, genital bleeding, hematuria, petechiae, buccal hematoma, and hemorrhagic blisters. Furthermore, most patients were treated with corticosteroids, intravenous immunoglobulin (IVIG), or a combination of both. One patient had a platelet count >100 × 10^9^/L, and no treatment was administered. The overall outcome of all patients was favorable, with no reported deaths. The detailed findings are outlined in Table 1 and Table 2.

Of the observational studies included, three each were conducted in France and the United States, two in Italy, and one each in Berlin, Japan, and Taiwan. A case–control analysis was additionally performed within one of the three self-controlled case series studies, and one study included three different age groups. Ten studies were conducted between 2000 and 2018. Based on the methodological quality score, all 10 studies had NOS scores ≥ 7 and were deemed high quality. The details are presented in Table 3.

### 3.3. Results of Meta-Analysis

**Figure 2:**
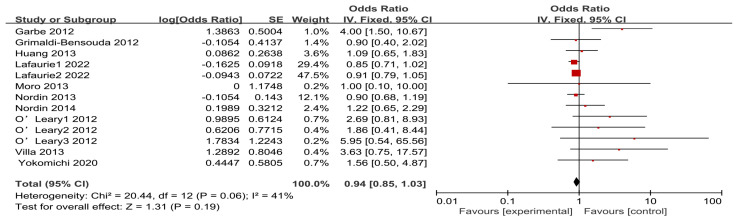
Forest plot of the association between influenza vaccination and ITP. Black diamonds: overall effect size and its 95% confidence interval. Red graph: effect size of each study and its weight.

The studies included in the analysis exhibited low heterogeneity and were pooled using a fixed-effects model. The meta-analysis found no evidence of an association between influenza vaccination and ITP, with a pooled OR of 0.94 (95% CI: 0.85–1.03). The results of the meta-analysis are presented in Figure 2. To further determine whether there were differences between the study designs, vaccine types, and ITP occurrence, we conducted subgroup analyses. No significant associations were observed across different study designs. The ORs for case–control, cohort, and self-controlled case series studies were 0.91 (95% CI: 0.76–1.07), 0.98 (95% CI: 0.76–1.26), and 0.94 (95% CI: 0.83–1.08), respectively. When studies were grouped by type of influenza vaccine, no significant correlations were identified between monovalent (OR = 1.14, 95% CI: 0.77–1.68) and trivalent (OR = 1.03, 95% CI: 0.79–1.34) influenza vaccines. The outcomes of the subgroup analysis are presented in Table 4.

### 3.4. Publication Bias

**Figure 3:**
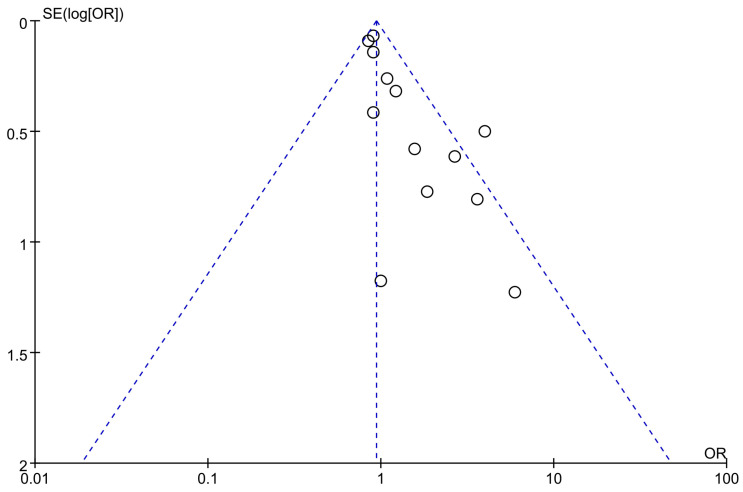
Funnel plot for the assessment of publication bias.

Figure 3 shows the funnel plot generated for this study. An examination of the plot reveals asymmetry, which suggests the potential presence of publication bias. This finding is further supported by the results of Egger’s and Begg’s tests, both of which indicated a statistical indication of publication bias within the included studies.

### 3.5. Sensitivity Analysis

**Figure 4:**
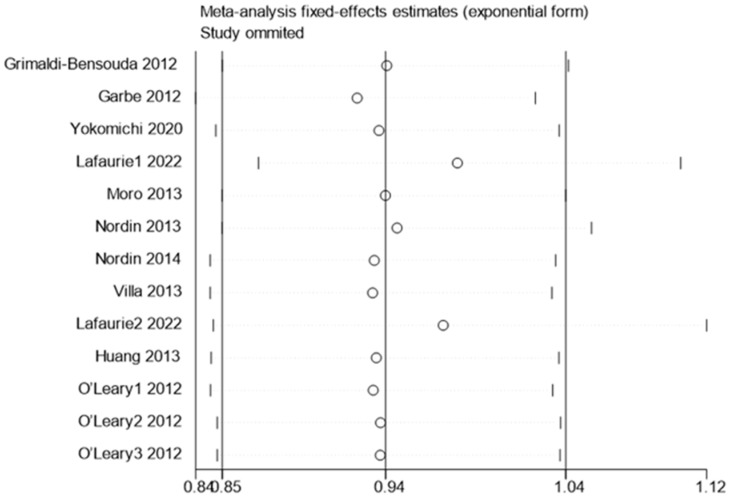
Sensitivity analysis of influenza vaccination and the risk of ITP. 0.85–1.04 represents the 95% confidence interval for the pooled effect size. The circle represents the effect size after removing the study, and the two short vertical lines in the same row as the circle represent the 95% confidence interval of the effect size after removing the study.

The sensitivity analysis results showed no significant change in the combined effect values before and after the study-by-study exclusion, indicating good robustness and reliability of the findings. This is illustrated in Figure 4.

## 4. Discussion

Whether ITP development is associated with influenza vaccination remains a topic of debate. Therefore, conducting a systematic review and meta-analysis of pertinent studies is necessary to thoroughly evaluate this relationship. Our study identified 16 patients who developed ITP after influenza vaccination. However, the aggregated findings from the meta-analysis did not reveal a statistically significant association between influenza vaccination and an increased risk of ITP development. These results suggest that although individual cases have been reported, the overall evidence fails to support a causal link.

The diagnosis of ITP was exclusionary and involved a detailed history, physical examination, and peripheral blood smear [47]. All the above cases were caused by the influenza vaccine after ruling out other potential causes. In this study, the mean age of patients with ITP secondary to influenza vaccination was found to be 56.7 years, similar to that observed in a case–control surveillance study in Berlin, where the median age of patients with drug-induced ITP was 56 years [23]. Other epidemiologic studies have also shown that the disease peaks in older patients [48]. In addition to the increased incidence of ITP with age, this occurrence may be attributed to the fact that influenza vaccination is recommended to the older population, and they are more likely to have bleeding manifestations. However, we were unable to determine sex differences in patients with influenza vaccine-associated ITP because of the insufficient number of cases. Recent findings reported a higher prevalence in females, correlating with an increased incidence of systemic autoimmune diseases in women [49]. Of note, one patient reported by Hamiel et al. had been hospitalized twice for ITP, with both instances occurring within one week of influenza vaccination [33]. This seems to reinforce the causal relationship between the influenza vaccine and ITP, but some studies have proposed that the influenza vaccine may trigger and worsen pre-existing thrombocytopenia [50]. Diabetes mellitus, hypertension, thyroid disease, and gastrointestinal disorders are the most common comorbidities in adult patients with ITP [51]. In our study, it was similarly found that some patients had diabetes mellitus, hypertension, and chronic angina prior to vaccination, suggesting that the development of ITP is not associated with chronic diseases. Only two patients took longer than one month from diagnosis to recovery, which may have been caused by their treatment strategies and physical conditions. Corticosteroids and IVIG are the first-line treatment options for ITP, and a combination of the two can accelerate outcomes [52]. In this study, most of the patients showed improvement in platelet counts and bleeding manifestations after using corticosteroids and IVIG. Although IVIG is less effective in older adults and has potentially serious side effects, it remains the best treatment in emergency situations [53]. It is important to note that most ITP cases are asymptomatic, leading to other potential undetected occurrences.

The current meta-analysis found no association between influenza vaccination and ITP risk. Subgroup analyses of different study designs and vaccine types yielded similar results. However, this finding contradicts the results of a previous meta-analysis. In Elsaid et al.’s study, the IRR of ITP 42 days after influenza vaccination was 1.85 (95% CI: 1.03–3.32) [54]. This result was derived by combining three different age subgroups from one study; however, our study included more studies with different designs and vaccination times. The results of our study showed that neither monovalent nor trivalent influenza vaccines increased the risk of ITP. In a comparative study assessing the safety of MF59 adjuvanted trivalent influenza vaccines in older adults, Villa et al. reported a 4.52-fold increased risk of ITP after adjuvanted trivalent influenza vaccination compared to the non-adjuvanted influenza vaccine. Therefore, further studies are required to confirm whether adjuvants affect the development of ITP. In addition, Yokomichi et al. explored the risk of ITP after inactivated, live, and concurrent vaccinations and found no significant risk of ITP after vaccination alone or concurrently in any age group.

The pathogenic mechanisms underlying vaccine-related ITP remain unclear. Current hypotheses primarily include molecular mimicry, bystander activation, epitope spreading, and polyclonal activation [55]. Among these, molecular mimicry is the most widely supported theory. This posits that due to structural similarities between epitopes, antibodies targeting antigens from infectious agents may cross-react with antigens on the platelet surface. Hemagglutinin is the main antigen of the influenza vaccine and shares structural similarities with platelet antigens [56]. Consequently, ITP triggered by the influenza vaccine may arise from Hemagglutinin interacting with platelets through certain receptors, promoting platelet lysis. Additionally, adjuvants, yeast proteins, and preservatives present in the influenza vaccine could activate polyclonal B cells, enhancing autoimmune responses and potentially inducing ITP or other autoimmune diseases. Some studies have shown that directly adding the H3N2 virus to platelets induces platelet aggregation in humans and animals, suggesting that the influenza virus may also directly affect platelets [57].

Our study has some limitations. First, we searched three databases and included only English literature, which may have led to the omission of some important studies. Future studies could expand the search and inclusion criteria to obtain more relevant data. Second, owing to the limited data in the articles included in the study, we were unable to analyze subgroups according to age and vaccination duration. Third, two of the studies included pregnant women as participants; therefore, our findings did not allow for an accurate assessment of the risk of ITP in the whole population. Fourth, most patients with ITP are asymptomatic, leading to underreporting; therefore, in this study, we may have missed this subset of cases. Finally, our study had a publication bias, which may affect the reliability of our findings, but we deem that it was not caused by the sample size or quality of the study. Several abstracts and conference papers with relevant data were found in the literature search, but we did not include them in the study. It is unclear whether including these studies would reduce publication bias. Additionally, including non-English studies and unpublished data may also address this bias. Thus, the findings of this study should be approached and interpreted with caution.

## 5. Conclusions

In this systematic review and meta-analysis containing 24 studies, we found a low probability of developing ITP following influenza vaccination, along with generally favorable prognostic outcomes. This study provides no evidence to support a causal relationship between influenza vaccination and immune thrombocytopenia. Therefore, while maintaining the current influenza vaccination strategies, the monitoring of abnormal vaccination reactions should be further enhanced. Furthermore, larger observational studies are required to more accurately assess the potential relationship between ITP and influenza vaccination. More longitudinal and diverse research across varied communities is needed to investigate the long-term consequences and potential demographic disparities in immunological responses. Future research should look into the function of adjuvants or other components in vaccinations and their possible impact on immune response and autoimmune consequences. In addition, the development of ITP after other vaccinations has been reported in several studies, and it is equally interesting to ponder whether there is an association between them. This area of research warrants further investigation to enhance our understanding of vaccine-related adverse events and inform vaccination practices.

## Figures and Tables

**Table 1:** Characteristics of the included case reports.

Study	Age (Years)	Sex	Vaccine Dose	Platelet Count		Time from	
				Before Vaccination	After Vaccination	Vaccination to Diagnosis	Recovery from ITP
Shlamovitz 2013 [25]	50	M	1	NA	<5 × 10^3^/μL	4 d	6 d
Mantadakis 2010 [26]	3	M	2	NA	11 × 10^3^/μL	26 d	1 d
Asiimwe 2023 [27]	70	M	NA	Normal	2 × 10^3^/μL	6 d	3 d
Almohammadi 2019 [28]	68	M	3	210–267 × 10^3^/μL	0/μL	2 d	10 d
Cummins 1998 [29]	67	F	NA	NA	111 × 10^9^/L	21 d	14 d
Ikegame 2006 [30]	19	F	1	>180 × 10^9^/L	10 × 10^9^/L	14 d	19 d
Tishler 2006 [31]	68	M	NA	NA	3000/mm^3^	14 d	5 d
Nagasaki 2016 [32]	81	F	NA	184 × 10^3^/μL	39 × 10^3^/μL	28 d	Within 5 months
	75	F	NA	251 × 10^3^/μL	5 × 10^3^/μL	35 d	28 d
	87	F	NA	NA	2 × 10^3^/μL	14 d	NA
Hamiel 2016 [33]	4.5	M	3	>200 × 10^3^/μL	17 × 10^3^/μL	6 d	10 d
Ohta 2022 [34]	88	M	NA	150 × 10^3^/μL	1 × 10^3^/μL	4 d	NA
Mamori 2008 [35]	75	F	NA	164 × 10^3^/μL	5 × 10^3^/μL	7 d	16 d
Wan Jamaludin 2018 [36]	31	F	1	203 × 10^9^/L	3 × 10^9^/L	7 d	1 year
Kelton 1981 [37]	38	M	NA	Normal	20 × 10^3^/μL	14 d	NA
Mateos 2007 [38]	83	F	NA	NA	1 × 10^9^/L	10 d	5 d

M, Male; F, Female; NA, Not Available; d, day.

**Table 2:** Clinical characteristics of the patients in the case reports.

Study	Medical History	Presentation	Treatment	Outcome
Shlamovitz 2013 [25]	None	Bleeding gums	Prednisone and IVIG	Recovery
Mantadakis 2010 [26]	None	Diffuse petechiae	IVIG	Recovery
Asiimwe 2023 [27]	HP infection	Mixed petechial rash, oral bleeding	Methylprednisolone, IVIG, oral dexamethasone	Recovery
Almohammadi 2019 [28]	Hepatitis C, prediabetes, hypertriglyceridemia	Epistaxis, hematuria, and bleeding gums	Platelet transfusion, IVIG, dexamethasone, and oral prednisone	Recovery
Cummins 1998 [29]	Chronic angina	Abnormal blood count	No treatment	Recovery
Ikegame 2006 [30]	Acute lymphoblastic leukemia	Transient nasal bleeding	Immunoglobulin, and prednisolone	Recovery
Tishler 2006 [31]	Hypertension	Rash and melena	Intravenous gamma globulins, corticosteroids, red blood cells, and proton pump inhibitors	Recovery
Nagasaki 2016 [32]	None	None	HP eradication	Recovery
	None	Gingival and nasal hemorrhaging	HP eradication, prednisolone	Recovery
	None	genital bleeding, purpura	Prednisolone	Recovery
Hamiel 2016 [33]	None	Cutaneous and mucosal bleeding	IVIG	Recovery
Ohta 2022 [34]	Brain stroke, hypertension, and dyslipidemia	Oral bleeding, bleeding blisters, and systemic petechiae	Prednisolone	Recovery
Mamori 2008 [35]	Primary biliary cirrhosis	Epistaxis, petechiae	Prednisone	Recovery
Wan Jamaludin 2018 [36]	Hodgkin Lymphoma	Spontaneous bruises, petechiae, and buccal hematoma	Corticosteroids with a slow tapering course of prednisolone	Recovery
Kelton 1981 [37]	Chronic obstructive pulmonary disease, bronchiectasis	Purpura and hemoptysis	Corticosteroid	Recovery
Mateos 2007 [38]	Chronic lymphocytic leukemia	Petechiae, bruising, and hemorrhagic bullae in oral mucosa	Methylprednisolone	Recovery

HP, Helicobacter pylori; IVIG, Intravenous immunoglobulin.

**Table 3:** Characteristics of observational studies.

Study	Study Design (Period)	Area	Age	Vaccine Type	OR	95%CI	NOS
Grimaldi-Bensouda 2012 [22]	Case-control (2008–2011)	France	18–79	All	0.9	0.4–2.1	9
Garbe 2012 [23]	Case-control (2000–2009)	Berlin	≥18	All	4.0	1.5–9.6	9
Yokomichi 2020 [39]	Case-control (2015–2017)	Japan	NR	All	1.56	0.50–4.85	8
Lafaurie1 2022 [40]	Case-control (2009–2018)	France	≥65	All	0.85	0.71–1.03	7
Moro 2013 [41]	Cohort (2009–1010)	Italy	All	MIV	1	0.1–16.0	7
Nordin 2013 [42]	Cohort (2002–2009)	US	NR	TIV	0.9	0.68–1.19	7
Nordin 2014 [43]	Cohort (2009–2010)	US	NR	MIV	1.22	0.65–2.28	7
Villa 2013 [44]	Cohort (2006–2009)	Italy	≥65	TIV	3.63	0.75–10.62	7
Lafaurie2 2022 [40]	Self-controlled case series (2009–2018)	France	≥65	All	0.91	0.79–1.05	7
Huang 2013 [45]	Self-controlled case series (2009–2010)	Taiwan	All	MIV	1.09	0.65–1.85	7
O’Leary1 2012 [46]	Self-controlled case series (2000–2009)	US	6–23 months	TIV	2.69	0.81–8.88	
O’Leary2 2012 [46]			2–6	TIV	1.86	0.41–8.38	7
O’Leary3 2012 [46]			7–17	TIV	5.95	0.54–65.96	

NR, No Reported; MIV, Monovalent Influenza Vaccines; TIV, Trivalent Influenza Vaccines.

**Table 4:** Results of the subgroup analysis.

Subgroups	Numbers of Studies	I^2^ (%)	OR (95% CI)	*p*-Value
Study design				
Case-control	4	71	0.91 (0.76–1.07)	0.26
Cohort	4	14	0.98 (0.76–1.26)	0.87
Self-controlled case series	5	39	0.94 (0.83–1.08)	0.41
Vaccine type				
All	5	61	0.91 (0.81–1.01)	0.08
TIV	5	53	1.03 (0.79–1.34)	0.82
MIV	3	0	1.14 (0.77–1.68)	0.52

MIV, Monovalent Influenza Vaccines; TIV, Trivalent Influenza Vaccines.

## Data Availability

All studies we included were searchable on Web of Science, Embase, or PubMed databases.
